# Hyperresponsiveness to antihistamines in spontaneous urticaria and heat urticaria 

**DOI:** 10.5414/ALX01554E

**Published:** 2017-08-04

**Authors:** J. Durda, B. Wedi, V. Martin, K. Breuer

**Affiliations:** 1 Abteilung für Allergologie und Berufsdermatologie, Dermatologikum Hamburg and; 2 Klinik für Dermatologie, Allergologie und Venerologie, Medizinische Hochschule Hannover, Germany

**Keywords:** H1 antihistamine, urticaria, drug hypersensitivity, H1 receptor, CAST, FlowCAST

## Abstract

Abstract. Background: H_1_ antihistamines are important drugs for the treatment of urticaria and are commonly well tolerated. Cases of hypersensitivity reactions to antihistamines have rarely been reported, the underlying pathomechanism is unknown yet. Case report: We report the case of a 28-year-old female patient suffering from chronic spontaneous urticaria who experienced severe episodes of wheals and flares induced by different H_1_ antihistamines. Methods: We performed skin prick tests (SPT) with a diversity of H_1_ antihistamines and CAST and FlowCAST analyses. Moreover, a placebo controlled oral challenge test to mizolastin was done. Results: We saw positive SPT reactions to nearly all H_1_ antihistamines tested with the exception of mizolastin. We observed neither a release of sulfidoleukotrienes nor an upregulation of basophil activation markers in the CAST and FlowCAST analyses. The oral challenge test with mizolastine resulted in the development of generalized wheals and flares. Conclusion: H_1_ antihistamines are effective drugs for the treatment of urticaria, but they may lead to hypersensitivity reactions in rare cases. Because of the negative CAST and FlowCAST results, an IgE-mediated pathomechanism is improbable. We propose that hypersensitivity reactions to H_1_ antihistamines may be induced by a paradox H_1_ receptor activation.

German version published in Allergologie, Vol. 36, No. 4/2013, pp. 164-169

## Introduction 

H_1_ antihistamines have been used in therapy of urticaria for years and are usually very well tolerated. Histamine is a mediator that acts by binding to four different receptors (H_1_, H_2_, H_3_, H_4_ receptor). 

Histamine receptors are heptahelical transmembrane proteins transmitting extracellular signals to intracellular second-messenger systems (Ca^2+^, cGMP, NF-κB, and other) via G-proteins [1]. Histamine receptors are constitutively activated and can – even when no histamine is present – transmit signals from outside into the cell; active and inactive conformations of the receptors are balanced. H_1_ antihistamines consist of a single heterocyclic aromatic ring. Most antihistamines contain an ethyl amine group (X-C-C-N) that is also present in histamine. The therapeutic mechanism of H_1_ antihistamines is based on the inhibition of the effects of histamine mediated by H_1_ receptors. H_1_ antihistamines act as inverse agonists; the receptor is stabilized by the binding of antihistamine in its inactive conformation, and the balance is shifted towards the inactive state. 

In the literature, there are only few case reports describing hypersensitivity reactions to antihistamines. The underlying pathomechanisms are not yet understood. 

## Case report 

We report the case of a 28-year-old female patient who presented with a 4-year history of almost daily episodes of urticarial skin lesions. She reported to not yet be suffering from angioedemas. The patient suspected that the skin reactions were triggered by exposure to the sun. Furthermore, the intake of cetirizine tablets had led to a worsening of the urticaria in the past, and sometimes she had experienced episodes of vertigo and nausea after intake. Thus, the patient had self-tested the tolerability of several antihistamines during symptom-free periods. She reported always having developed urticarial lesions approximately 2 hours after intake. The triggering drugs were Rupafin^®^ (rupatadine), Telfast^®^ (fexofenadine), Atarax^®^ (hydroxyzine), Lorano^®^ (loratadine), Wick Medi Nait^®^ (among others, doxylamine succinate), and Grippostad^®^ (among others chlorpheniramine maleat). Furthermore, the patient indicated that Livocab^®^ (levocabastine) eye and nose drops, used for the treatment of rhinoconjunctival conditions, had also led to urticarial lesions in the entire integumentary system, mostly in the face. Her patient history included neurodermitis in childhood, rhinoconjunctival symptoms in the months of April to October, oral allergy syndrome after ingestion of apples and various nuts as well as perennial bronchial asthma. Due to migraines, she took acetylsalicylic acid and ibuprofen as needed, the latter twice a week. Due to relapsing tonsillitis, a tonsillectomy had been carried out, but after that, increased CRP levels had still repeatedly been measured. On the day of first presentation, the skin symptoms were weak, no urticarial lesions or red dermographism were detected. The physical examination revealed remnants of the tonsils at the soft palate. 

## Methods 

For work-up of possible triggering factors of chronic urticaria, several laboratory examinations were carried out: routine lab tests, thyroid hormones, thyroid autoantibodies, antinuclear antibodies, and throat swab. Additional physical tests were carried out: forearm bath at 40 °C, phototesting with a maximum of 0.15 J/cm^2^ UVB, 80 J/cm^2^ UVA). 

Allergologic tests using antihistamines were carried out according to the current recommendations for the diagnostic work-up of drug hypersensitivity [2, 3, 4]. Prick tests with various antihistamines were carried out. For this, antihistamine tablets were ground, 0.9% NaCl solution was added, and the supernatant as well as 10-vol% of the supernatant were dissolved in 0.9% NaCl solution and tested. The following drugs were tested: Telfast^®^ tablets (180 mg terfenadine), Rupafin^®^ tablets (10 mg rupatadine), Atarax^®^ tablets (25 mg hydroxycine), Lorano^®^ tablets (10 mg loratadine), Cetirizin ratiopharm^®^ tablets (10 mg cetirizine), Aerius^®^ coated tablets (5 mg desloratadine), Ebastel^®^ coated tablets (10 mg ebastine), Tavegil^®^ tablets (1 mg clemastine fumarate), Mizollen^®^ coated tablets (10 mg mizolastine), and Xusal^®^ coated tablets (5 mg levocetirizine). Livocab^®^ eye drops (levocabastine) were used in undiluted and diluted (10-vol% in 0.9% NaCl solution) form. The tests were read after 20 minutes and 24 hours. 

A single-blind placebo-controlled oral challenge with mizolastine was carried out during a symptom-free period. First, the patient received a placebo capsule, then, at intervals of 2 hours, 1 mg and later 5 mg of mizolastine in capsule form were administered, corresponding to 10% and 50% of the single dose, respectively. 

For CAST^®^ ELISA and FlowCAST^®^ (Bühlmann Laboratories, Schönenbuch, Switzerland), Fenistil^®^ injectable solution (dimethindene maleate), Tavegil^®^ injectable solution (clemastine fumarate), Cimetidin CT^®^ injectable solution, and Cetirizin Hexal^®^ drops (concentration of active substance 20 µg/mL and 200 µg/mL, respectively) were used. The tests were carried out according to manufacturer’s instructions. 

## Results 

Laboratory work-up: antibodies against thyroperoxidase 56 U/mL (normal value < 34 U/mL), euthyroid metabolic state corresponding to Hashimoto autoimmune thyropahty. Otherwise, laboratory values were inconspicuous. The tonsil swab showed beta-hemolyzing group C streptococci. A 20-minute forearm bath in warm water (40 °C) led to the development of areas of urticarial lesions in the contact area. Phototesting did not reveal any pathologic finding. 

The 20-minute reading of the skin prick test showed positive reactions (+ to ++) to all undiluted antihistamines, except mizolastine. In the skin prick test, the 1 : 10 solutions were negative for loratadine, desloratadine, ebastine, and mizolastine; all other antihistamines showed positive (+) test reactions (the negative control was inconspicuous). At 24-hour reading, no skin symptoms were visible. 

**Figure 1.**
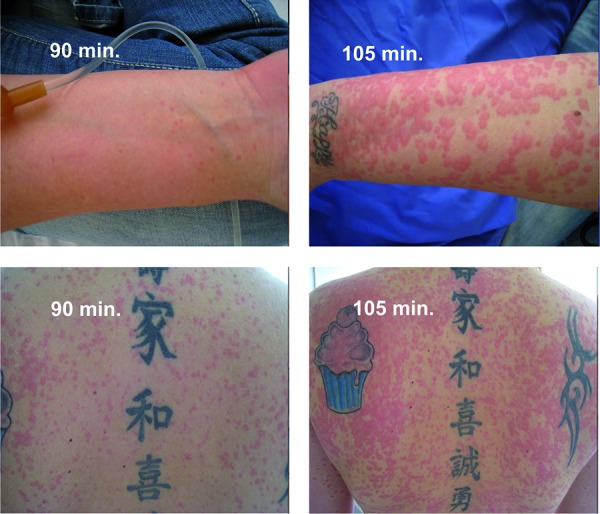
Single-blind, placebo-controlled oral challenge test with mizolastine. Development of generalized urticarial skin reaction starting 90 minutes after the intake of 5 mg mizolastine.

As no reaction to mizolastine was observed in the skin prick test, oral challenge with Mizollen^®^ was carried out. 90 minutes after the administration of 5 mg mizolastine, pinhead-sized urticarial lesions were seen on the entire body; later, the lesions increased and coalesced (Figure 1). Further symptoms were not observed. For therapy, 125 mg Solu Decortin H was administered intravenously, and the lesions slowly improved after 3 hours. 

The antihistamine concentrations used in the CAST did not lead to a de-novo sulfidoleukotrien production, and no upregulation of CD63 was observed in the FlowCAST. Our first recommendation was to stop ibuprofen and acetylsalicylic acid intake. The migraine medication was changed to paracetamol and rizatriptan by the patient’s neurologist. Furthermore, we recommended not using H_1_ antihistamines in the future. In the case of urticaria episodes, we recommended she use low-dose oral glucocorticosteroids (up to 20 mg prednisolone equivalent) instead. 

As beta-hemolyzing group C streptococci were found in the tonsil swab, antibiotic therapy with Isocillin 3-times 1.2 Mega was carried out for 10 days. During a symptom-free interval, the tonsil remnants were removed operatively. Six weeks after tonsillectomy, the patient reported significantly less events of urticaria, mainly induced by warmth. Three months later, urticarial lesions only appeared from time to time. The patient refused systemic therapy with, e.g., dapsone, cyclosporine, or omalizumab. 

## Discussion 

We report a patient who experienced aggravation of urticaria (chronic spontaneous urticaria and heat urticaria) after intake of various H_1_-antihistamines. Skin prick tests were mainly positive; the single-blind, placebo-controlled oral challenge test with mizolastine, the only H_1_-antihistamine that did not cause a reaction in the skin test, led to the development of generalized wheals and flares approximately 90 minutes after administration of 50% of the single dose (5 mg). 

Adverse reactions to antihistamines have been reported in the literature. Rodríguez de Río et al. [5] reported five cases of urticarial skin reactions after the intake of H_1_-antihistamines, which had been administered due to acute urticaria or allergic rhinoconjunctivitis. In 4 of these patients, the skin reactions occurred approximately 2 – 3 hours after antihistamine administration; in 1 female patient, the skin reaction developed only 24 hours after the intake of the drug. In 4 of these patients, all skin prick tests using various antihistamines of all classes were negative. Nevertheless, oral challenge testing with the negatively tested antihistamines could induce skin reactions in these patients. In all of these cases, at least one antihistamine could be found that was tolerated by the patient. Similar to our case, positive skin prick test reactions to antihistamines were seen in 1 female patient. This patient reacted to all antihistamines with urticarial skin symptoms after approximately 2 – 3 hours. Another Spanish case report describes a female patient with allergic rhinitis who developed urticarial skin lesions after oral provocation with various antihistamines, some of which had been tested negative in a skin prick test [6]. There are also several reports on single patients with chronic spontaneous urticaria who experienced an exacerbation of the skin disease after intake of first- or second-generation H_1_-antihistamines. In all cases, the skin symptoms appeared a few hours after intake [7, 8, 9]. 

In addition to skin symptoms, shock reactions have been reported as adverse reactions to antihistamines. For example, Barranco et al. [10] describe a female patient who developed urticarial skin lesions, angioedema, and dyspnea a few minutes after the intake of diphenhydramine, a first-generation H_1_-antihistamine. Intradermal testing with diphenhydramine was positive. A reaction in the skin prick test was also shown in a female patient who had taken mizolastine due to allergic rhinitis and experienced an anaphylactic reaction with generalized urticaria, dyspnea, and drop in blood pressure 2 hours later [11]. Intake of further first- and second-generation H_1_-antihistamines was tolerated in an oral challenge test. Our patient also reported vertigo and nausea after intake of cetirizine, but systemic reactions after the intake of other H_1_-antihistamines did not occur. 

The underlying pathomechanism of these reactions is currently not known. Some authors have suggested IgE-mediated reactions. By metabolization of antihistamines, cross-reactive metabolites could be generated that, similarly to beta-lactam antibiotics, act as haptens and induce the synthesis of specific IgE-antibodies. A primary involvement of the metabolites, but not of the antihistamines themselves, could explain why CAST and FlowCAST with various first- and second-generation H_1_-antihistamines were negative in our patient. CAST is a cellular in-vitro assay evaluating the de-novo production of sulfidoleukotrienes (LTC4, LTD4, LTE4) in leukocyte suspensions after stimulation. Leukocyte suspensions with the suspected allergen (in this case the antihistamine), enriched via dextrane sedimentation, are incubated. If hypersensitivity to the allergen/antigen is present, IgE-dependent, but also IgE-independent, mechanisms lead to the degranulation of basophil granulocytes and to the release of sulfidoleukotrienes, which can be demonstrated by ELISA. In the FlowCAST, CD63 surface molecules (markers of activation) on basophil granulocytes are upregulated after allergen binding; these CD63 surface molecules are quantified by flow cytometry. The hypothesis described above is contradicted by the fact that all first-generation and some second-generation (loratadine, desloratadine) H_1_-antihistamines are metabolized by the cytochrome P450 system (CYP450) in the liver, while cetirizine is excreted via the kidneys in an almost unmodified form, and fexofenadine is excreted in an unmetabolized form via the feces [1], and also the latter two led to hyperresponsiveness reactions in our case. With a clearly positive skin test, as was the case in our patient, the CAST/FlowCAST with cetirizine would at least have had to have been positive in the case of an IgE-mediated reaction to cetirizine. It might, however, be possible that the antihistamine concentrations used for cellular in-vitro diagnostic work-up were not ideal. 

H_1_-antihistamines act as inverse agonists and stabilize the H_1_-receptor in its inactive conformation, with the receptor balance being shifted towards the inactive state [1]. In our case, a paradoxical effect of H_1_-antihistamines can be discussed. In this case, the H_1_-receptor would be stabilized in its active conformation due to the binding of the antihistamine that possesses a high structural similarity to histamine. Possibly, this uncommon reaction to H_1_-antihistamines could be caused by polymorphisms of the H_1_-receptor. H_1_-recpetor polymorphisms have been described in asthma patients, but, to date, it is unclear how they influence the clinical effects of antihistamines [12]. The fact that various groups of H_1_-antihistamines – piperidines (fexofenadine, loratadine, desloratadine, ebastine, mizolastine) and piperazines (cetirizine, levocetirizine, and hydroxyzine) – led to skin reactions in our patient makes a paradoxical receptor activation appear plausible. 

In conclusion, we describe a female patient with chronic spontaneous and physical urticaria triggered not only by chronic infection and intake of NSAR, but also by the use of first- and second-generation H_1_-antihistamines. By avoiding the triggering factors and by treating the focus of infection, the urticaria symptoms almost completely resolved. To date, the underlying pathomechanisms of hyperresponsiveness reactions to antihistamines are still unclear.
